# Crural and Plantar Fasciae Changes in Chronic Charcot Diabetic Foot: A Cross-Sectional Ultrasound Imaging Study—An Evidence of Fascial Continuity

**DOI:** 10.3390/jcm12144664

**Published:** 2023-07-13

**Authors:** Carmelo Pirri, Carlo Biz, Nina Pirri, Veronica Macchi, Andrea Porzionato, Raffaele De Caro, Pietro Ruggieri, Carla Stecco

**Affiliations:** 1 Department of Neurosciences, Institute of Human Anatomy, University of Padova, 35121 Padua, Italy; veronica.macchi@unipd.it (V.M.); andrea.porzionato@unipd.it (A.P.); rdecaro@unipd.it (R.D.C.); carla.stecco@unipd.it (C.S.); 2 Department of Orthopedics and Orthopedic Oncology, University of Padova, 35128 Padova, Italy; pietro.ruggieri@unipd.it; 3 Department of Medicine—DIMED, School of Radiology, Radiology Institute, University of Padova, 35121 Padova, Italy; nina_92_@hotmail.it

**Keywords:** plantar fascia, ultrasound examination, thickness, diabetes, pain, crural fascia

## Abstract

Crural fascia (CF) and plantar fascia (PF) are biomechanically crucial in the gait and in the proprioception, particularly in the propulsion phase of the foot during the gait cycle and in the dissipation of forces during weight-bearing activities. Recent studies have revealed an association between increases in PF thickness and diabetes. The purpose of this study was to measure and compare by ultrasound (US) imaging the thickness of the CF and PF at different regions/levels in chronic Charcot diabetic foot patients (group 1) and in healthy volunteers (group 2). A cross-sectional study was performed using US imaging to measure the CF with Pirri et al.’s protocol and PF with a new protocol in a sample of 31 subjects (15 patients and 16 healthy participants). The findings for CF and PF revealed statistically significant differences in the poster region of CF (Post 1: group 1 vs. group 2: *p* = 0.03; Post 2: group 1 vs. group 2: *p* = 0.03) and in PF at two different levels (PF level 1: group 1 vs. group 2: *p* < 0.0001; PF level 2: group 1 vs. group 2: *p* < 0.0001). These findings suggest that chronic Charcot diabetic foot patients have CF and PF thicker compared to healthy volunteers. The US examination suggests that fascial thicknesses behavior in these patients points out altered fascial remodeling due to diabetes pathology and biomechanical changes.

## 1. Introduction

Diabetes mellitus (DM) is the “major metabolic epidemic” of the 21st century, and its prevalence continues to increase worldwide [1]. The International Diabetes Federation (IDF) reported that there are over 460 million adults in the world affected by this disease and that this number is destined to increase further [1]. Consequently, its complications increase in terms of prevalence with large growth in economic expenditure [1]. Among the latter, the diabetic foot is one of the main factors of morbidity and mortality associated with diabetes [2,3]. Indeed, it is estimated that 50% of hospitalizations related to diabetes are caused by consequential foot problems (infection, ulceration, osteomyelitis, etc.), halving survival at 1 and 5 years compared to other diabetic patients without the aforementioned alterations [4]. In general, the diabetic foot and its intra- and extra-hospital treatment are responsible for up to 20% of the economic expenditure for diabetes [5].

Despite having an important impact on the disability of diabetes patients, the mechanism of diabetic foot chronicity has not yet been understood in a complete way [6]. Factors associated with the pathogenesis of diabetic foot are complex and multifactorial but fundamentally involve the interaction of extrinsic biomechanical forces with intrinsic structural and functional properties of the soft tissues [7,8,9]. Charcot diabetic foot is a chronic complication with a unilateral onset that becomes bilateral over time, presenting a distinct evolution in two phases, acute and chronic [10]. The prevalence of Charcot foot in the diabetes population stands between 0.1% and 0.4% but rises up to 35% among diabetics who have developed advanced peripheral neuropathy [4,10,11]. Indeed, the latter is related to the diabetic foot due to the consequent alterations of sensitivity, motility, and autonomic processes of the anatomical foot structures [12,13,14,15,16,17,18,19,20,21]. Histological alterations have been observed in the soft tissues of the plantar region [22,23] as well as the Achilles tendon [24,25]. Moreover, the excessive accumulation of advanced glycosylation end-products (AGEs) has been implicated not only in muscles, nerves, skin, and tendons but also fasciae [26], encompassing various organs and tissues throughout the human body. Concurrent remodeling of the fascial tissue may lead to changes in mechanical behavior. When combined with other well-established risk factors, such as diabetes-related peripheral neuropathy (DPN) and foot deformities, this alteration in fascial tissue may increase the risk of diabetic-related foot ulceration (DFUs) [25,26].

The fascia, in terms of its structure, functions as a protective and functional covering that surrounds and separates muscles. It is significantly affected by the hormonal and endocannabinoid system [27,28,29], as well as mechanical and age factors [29]. In individuals with diabetes and foot complications, there is an increased stiffness of the tissue on the bottom of the foot, which is believed to disrupt the distribution of stretching/tensile forces and pressure/load within the soft tissues of the foot during walking [30]. This means that the repetitive biomechanical stresses that the foot normally absorbs during everyday activities may not be adequately dissipated, ultimately leading to the development of diabetic foot [25]. Additionally, it is important to note that plantar fascia (PF) is not an isolated structure; Stecco et al. [31] demonstrated its anatomical connection with AT and fascia of lower leg/crural fascia (CF).

Ultrasound (US) imaging is able to visualize the fascial layers [32] and has become important in fascia examination [33,34,35,36,37]. The fascia thickness is a parameter to be assessed during the US examination of fasciae. Different researchers have studied the problem of US plantar tissue thickness evaluation in diabetes patients [38,39,40,41,42,43]. The heterogeneity of methodological approaches and studies of the assessment of plantar fascia thickness made it difficult to compare. For this reason, the purpose of this cross-sectional study was to investigate the difference in plantar fascia thickness proximal to the calcaneus and distal with a new protocol among chronic Charcot diabetic foot patients and a healthy control group. Finally, we decided to evaluate the crural fascia to highlight any fascial changes in the leg.

Considering the biomechanical role of PF and CF, specifically their involvement in the foot’s propulsion phase of the foot during the gait cycle and in the distribution of forces during weight-bearing activities, the study’s hypothesis was to demonstrate an alteration in the thickness of both, creating a new US assessment protocol.

## 2. Materials and Methods

### 2.1. Study Design

A cross-sectional study based on the Strengthening the Reporting of Observational Studies in Epidemiology (STROBE) statement was conducted [44] in order to compare the US thickness of plantar fascia and crural fascia at different levels among chronic Charcot diabetic foot patients and healthy control group. The Helsinki Declaration and human experimentation rules [45,46] were considered, and the Ethics Committee approved the research (approval no. 3513/AO/15, study approved on 28 January 2016 by the Ethical Committee for clinical trials in the province of Padova). All of the participants were informed prior to inclusion in the project by being provided with a written consent form.

### 2.2. Participants and Clinical Assessment

A total sample of 31 subjects was recruited and divided into two groups: “group 1” comprised 15 subjects with chronic Charcot diabetic foot; and “group 2” comprised 16 healthy subjects, from October 2018 and June 2021. Based on the following criteria, the inclusion criteria for group 1 participation consisted of some parameters: patients with a clinical and radiographic diagnosis of diabetes complicated by chronic phase Charcot neuro-osteo-arthropathy (Eichenholtz stage 3) at the level of the foot, monoliteral or bilateral, confirmed by upright radiography, evaluated by experienced orthopedic surgeon. The exclusion criteria for group 1 included age > 75 years old, Charcot diabetic foot operated to correct deformities, previous orthopedic surgery of the lower limb, active foot ulcers, rheumatic and connective tissue diseases, patients with pancreas transplant, whose antidiabetic therapy has, therefore, been suspended, neoplastic patients. The healthy normoglycemic participants were recruited among relatives of doctors of the department and the hospital staff. The exclusion criteria for group 2 encompassed individuals with a documented medical history involving lower extremities surgery, foot deformities, pain in the lower limbs, a history of fracture of the lower extremities, fibromyalgia, balance disorders, and systematic disease, such as rheumatological conditions and diabetes, among others.

The subjects underwent a US examination to evaluate the US thickness of PF and CF. The recruitment of participants was carried out by an orthopedic physician specializing in diabetic foot conditions possessing over a decade of experience in the field.

The participants of both groups underwent the following clinical investigations: blood pressure was measured at ankles, arms, and ankle-brachial index (ABI). Finally, for group 1 were measured the neuropathy disability score (NDS) [44], neuropathy symptoms score (NSS) [44], 12-item short-form survey (SF-12) (available online: https://orthotoolkit.com/sf-12/, accessed on 7 October 2018), and the evaluation of tobacco and alcohol consumption.

### 2.3. Ultrasound Examination Measurements

Utilizing a high-resolution device (Edge II, Sonosite, FUJIFILM, Inc. 21919, Lexington, WA, USA) equipped with a probe frequency range of 6–15 MHz and boasting a screen resolution of 1680 × 1050 pixels, US images were obtained at the foot and the leg regions/levels following a predefined US scanning procedure. The US assessments were performed by a physician who specialized in physical and rehabilitation medicine, possessing 7 years of experience in skeletal muscle US examination and US examination of fasciae. A standardized protocol was developed and employed to evaluate the PF bilaterally, while for the CF, a protocol previously published by Pirri et al. [33] was used, excluding the assessment of the anterior level 3, posterior level 3, and lateral levels. “The US system was set to a conventional speed of ultrasound (c = 1540 m/s) commonly used in diagnostic US systems, operating in B-mode and providing a depth of 30 mm; to ensure optimal scans and minimize surface pressure, the sonographer applied an appropriate amount of gel. The probe was positioned on the skin with light pressure to avoid tissue compression while maintaining stable contact for consistent imaging” [34,35,36,37]. The sonographer followed the same protocol to ensure consistent quantification of each point in the PF and CF. The US beam was maintained perpendicular to the PF and CF to mitigate the anisotropy that typically affected them. The power and overall gain of the US machine were adjusted to optimize visualization of the fascial layers and obtain high-quality scans. The resulting US images were frozen and captured.

**Figure 1:**
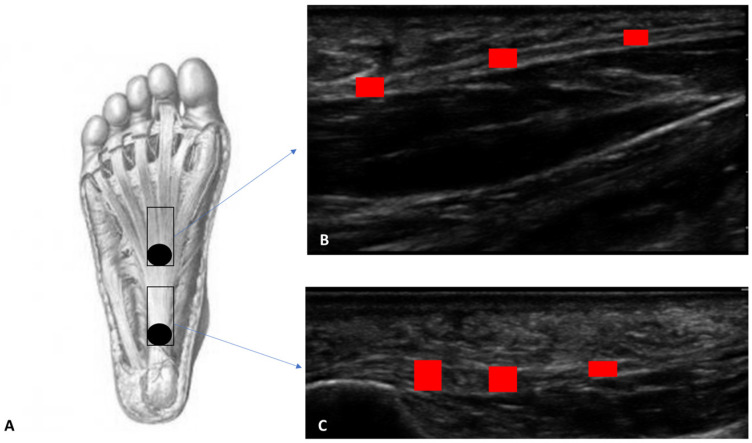
(**A**) Ultrasound measurement protocol of PF thickness at two levels of the plantar surface of the foot. The scans were taken on the long axis, paying close attention to maintaining the same structure in the center of the US monitoring image and keeping the probe perpendicular. (**B**) Level 1: at the calcaneal insertion of the PF up to 2 cm from it. (**C**) Level 2: in the middle third of the PF at 4–5 cm from the calcaneal insertion. Black circle: orientation of probe; red box: thickness of plantar fascia.

The sonographer used the short axis for the leg, according to Pirri et al. [33], whereas the PF used the longitudinal axis because, in the two topographical regions, they are the best axis to visualize and follow landmarks correlated with the fascial layers’ visualization imaging used by Pirri et al. [32]. A specific protocol for the PF was defined:

**PF:** the patient was relaxed in the prone position with the foot hanging freely over the edge of the examination table, maintaining the foot perpendicular to the leg and toes pointing down. The US transducer was placed longitudinally over the center arch of the foot. The US examination was performed at two levels: (level 1) at the calcaneal insertion of the PF up to 2 cm from it; (level 2) in the middle third of the PF at 4–5 cm from the calcaneal insertion. For this purpose, the probe was moved in proximal–distal direction (Figure 1). The scans were taken on the long axis, paying close attention to maintaining the same structure in the center of the US monitoring image and keeping the probe perpendicular.

At the conclusion of each assessment, all US images from every scan were saved and acquired. The measurement of fascial thickness was conducted using ImageJ analysis software (available online: https://imagej.nih.gov/iJ/, accessed on 5 March 2022). Each individual image was divided into three sections, and within each section, three points with the highest visibility were identified and measured. To mitigate the potential impact of thickness fluctuations, three equally spaced points were measured across the image, and the resultant values were averaged for further analysis. Moreover, the same procedure was repeated three different times to calculate the reliability of the measurements.

### 2.4. Statistical Analysis

Statistical analysis was performed using GraphPad PRISM 8.4.2. (GraphPad Software Inc., San Diego, CA, USA), and a *p* < 0.05 was always considered as the limit for statistical significance. The resulting effect size was calculated by G Power 3.1. (Universität Düsseldorf: Psychologie) and interpreted according to Cohen’s kappa as small (d = 20), medium (d = 0.50), and large (d = 0.80) [47]. Based on a first pilot study, the sample size calculated for both CF and PF was 7 subjects for the group, as the effect size was, respectively, for CF thickness d = 2 and for PF thickness d = 3.6, with α err prob = 0.05 and power: 1-β err prob = 0.95. Nevertheless, we could include a sample of 31 subjects in our group, a minimum of 15 subjects for the group.

The normality assessment was carried out using the Kolmogorov–Smirnov and Shapiro–Wilk tests. Descriptive and clinical statistics were calculated for both groups separately, including measures of central tendency and their dispersion ranges using mean and standard deviation (SD) to describe parametric data. Differences in US-estimated thickness of CF and PF across regions/levels were statistically analyzed by one-way analysis of variance (ANOVA) followed by Sidak’s multiple comparison test.

Finally, a comparative analysis between the chronic Charcot diabetic foot patient’s group and the healthy control group was performed using an unpaired Student’s *t*-test. In addition, Pearson’s test was employed for both groups to evaluate the correlation between the descriptive variables and US thicknesses.

Moreover, a two-way intra-class correlation coefficient (ICC 3,k) type C was used to assess the intra-rater reliability. The ICC values were interpreted as poor when below 0.5, moderate when between 0.5 and 0.75, good when between 0.75 and 0.90, and excellent when above 0.90 [48].

## 3. Results

A total of 31 subjects (17 females and 14 males) participated in this study. The descriptive data of the two groups are summarized in Table 1.

In regards to the characteristics of the chronic diabetic foot patients (group 1), only 2 cases out of 15 were affected by Diabetes Mellitus 1 (DM1) (13%), and 7 cases out of 15 were insulin dependent. The average duration of diabetes from diagnosis was 18.33 ± 12.15 years (range 6–47 years), while the glycemic control in only 1 out of 15 cases was adequate (5.99%). Eight out of 15 (53%) cases were affected by bilateral chronic Charcot diabetic foot (of the remaining case, four were right and three left). Regarding blood pressure, only 2 out of 15 (13%) patients were normotensive, and the remaining 13 out of 15 (87%) patients were affected by arterial hypertension. Finally, 7 out of 15 patients (47%) were affected by diabetic retinopathy (DR), and among them, 3 (20%) were also affected by diabetic nephropathy (DN), which was not detected in the absence of retinopathy (Table 2 and Table 3).

### 3.1. Clinical Assessment

#### 3.1.1. Group 1

The clinical assessments regarding peripheral neuropathy, peripheral arteriopathy, and other risk factors (cigarette smoke and alcohol consumption) of group 1 are reported in Table 4.

In group 1, ABI was, respectively, 0.94 ± 0.08 (range: 0.79–1.07) for the right and 0.96 ± 0.16 (range: 0.67–1.09) for the left. Vasculopathic limbs were 9 of 30, while 7 out of 23 chronic Charcot feet (30.4%) were complicated by peripheral vascular disease (Table 5). NDS values ranged from 3 to 10 (maximum score of 10), and Neuropathy Symptom Score values ranged from 0 to 8 points (maximum score of 9). All patients had a combination of NDS and NSS scores, distinguishing them as neuropathic (Table 4).

#### 3.1.2. Group 2 (Healthy Volunteers)

Regarding Table 6, only 1 volunteer reported an ABI value < 0.90. The average value of the ABI, respectively, of right and left were 1.01 ± 0.06 (range: 0.84–1.10) and 0.99 ± 0.06 (range: 0.84–1.09).

### 3.2. Ultrasound Measurements of the Crural and Plantar Fasciae

#### 3.2.1. Group 1 (Chronic Charcot Diabetic Foot)

Regarding Table 7, at Ant 1 and Ant 2, the CF in the chronic Charcot diabetic foot patients had, respectively, a mean US thickness of 0.75 ± 0.34 mm (Ant 1) and 0.71 ± 0.3 mm (Ant 2), while in the posterior region, CF had, respectively, a mean of US thickness of 1.24 ± 0.31 mm (Post 1) and 1.30 ± 0.30 mm (Post 2) (Table 7). Moreover, the US thickness of PF was, respectively, at level 1 (proximal) of 3.72 ± 0.70 mm while at level 2 (middle third) of 1.96 ± 0.43 mm (Table 7).

#### 3.2.2. Group 2 (Healthy Volunteers)

In the healthy volunteers, at anterior levels of the leg, the CF had, respectively, a mean US thickness of 0.72 ± 0.14 mm (Ant 1) and 0.76 ± 0.14 mm (Ant 2); while in the posterior region, CF had, respectively, a mean of US thickness of 0.97 ± 0.2 (Post 1) and 1.02 ± 0.30 (Post 2) (Table 8). Moreover, the US thickness of PF was, respectively, at level 1 (proximal) of 1.8 ± 0.57 while at level 2 (middle third) of 1.03 ± 0.42 mm (Table 8).

### 3.3. Ultrasound Measurements of Crural and Plantar Fasciae: Comparison between Group 1 and Group 2

**Figure 2:**
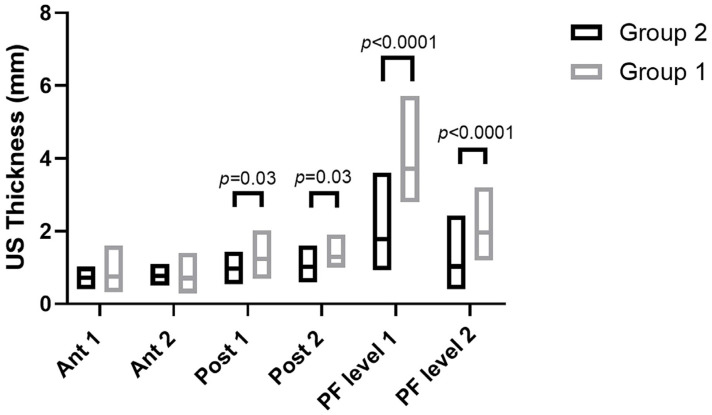
Ultrasound thickness measurements of crural and plantar fasciae in groups 1 and 2 at the different regions/levels.

**Figure 3:**
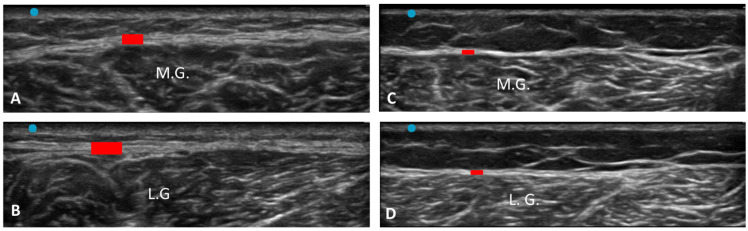
Ultrasound images of Crural fascia thickness: (**A**) group 1: Post 1 level of the leg according to Pirri et al. [33]; (**B**) group 1: Post 2 level of the leg according to Pirri et al. [33]; (**C**): group 2: Post 1 level of the leg according to Pirri et al. [33]; (**D**) group 2: Post 2 level of the leg according to Pirri et al. [33]. M.G.: Medial gastrocnemius muscle. L.G.: lateral gastrocnemius muscle. Red rectangles: crural fascia.

**Figure 4:**
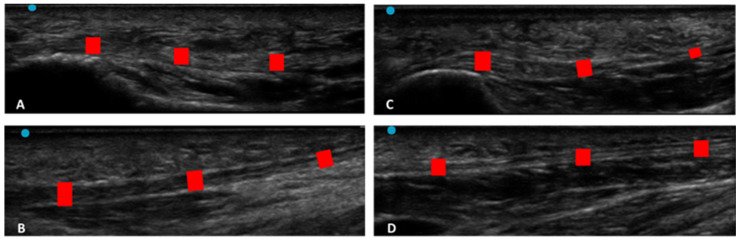
Ultrasound images of plantar fascia thickness: (**A**) group 1: PF level 1; (**B**) group 1: PF level 2; (**C**): group 2: PF level 1; (**D**) PF level 2. Red rectangles: plantar fascia.

According to Sidak’s test, the comparisons between the different regions/levels of the CF and PF between group 1 and group 2 showed a statistically significant difference in the US thickness: Post 1 (group 1 vs. group 2: *p* = 0.03), Post 2 (group 1 vs. group 2: *p* = 0.03), PF level 1 (group 1 vs. group 2: *p* < 0.0001) and PF level 2 (group 1 vs. group 2: *p* < 0.0001) (Table 9 and Figure 2, Figure 3 and Figure 4).

### 3.4. Correlation Ultrasound Measurements and Descriptive/Clinical Data

#### 3.4.1. Correlation Ultrasound Thicknesses and Years of Diabetes

**Figure 5:**
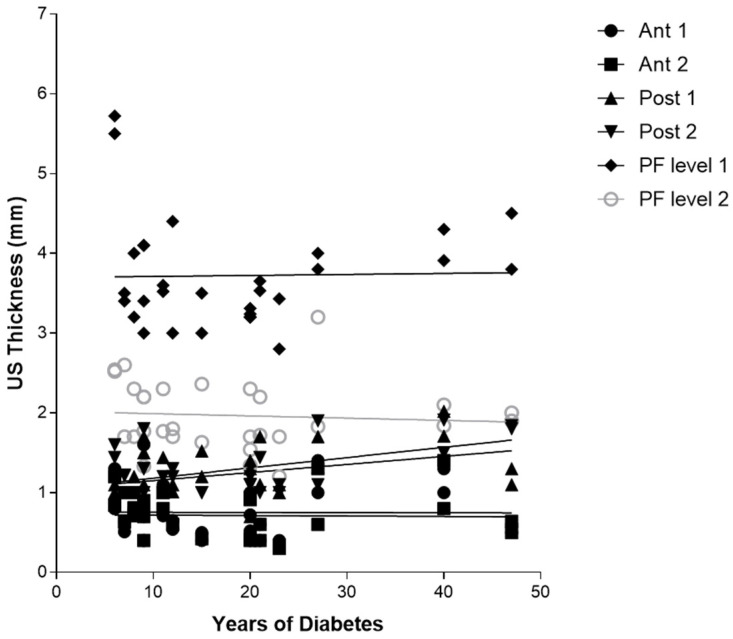
Correlation (Pearson R coefficient test) between fascial Ultrasound thickness and years of diabetes in group 1. Crural fascia: Ant1, Ant 2, Post 1, and Post 2; Plantar fascia: PF level 1 and PF level 2.

Regarding Table 10, there was no detected statistically significant correlation between the duration of diabetes in years and US thicknesses of Ant 1, Ant 2, PL level 1, and level 2, whereas Post 1 and Post 2 showed both a statistically significant correlation with the years of diabetes, respectively, for Post 1 (r = 0.3875, *p* = 0.0344) and for Post 2 (r = 0.5089, *p* = 0.0041) (Table 10 and Figure 5).

#### 3.4.2. Correlation Ultrasound Thicknesses and HbA1c

**Figure 6:**
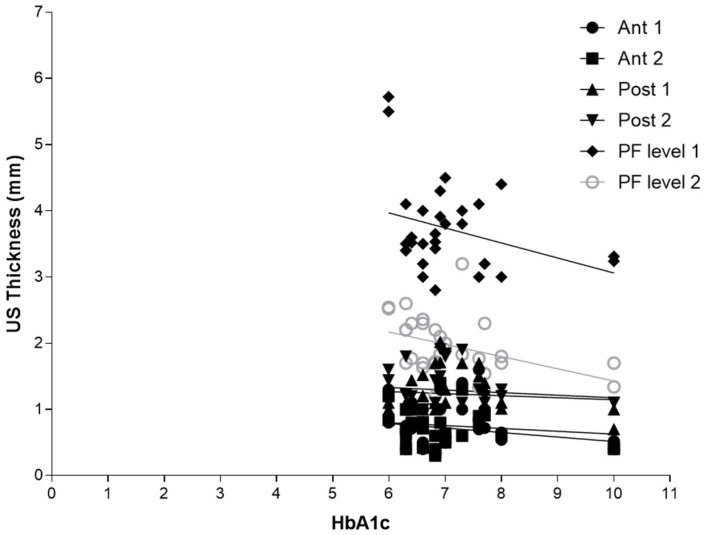
Correlation (Pearson R coefficient test) between fascial ultrasound thickness and HBA1c. Crural fascia: Ant1; Ant 2; Post 1; and Post 2. Plantar fascia: PF level 1; and PF level 2.

Regarding Table 11, there was no detected statistically significant correlation between the duration of diabetes in years and US thicknesses of Ant 1, Ant 2, Post 1, Post 2, and PL level 1, whereas PL level 2 showed a statistically significant correlation with the HbA1c (r = −0.4115, *p* = 0.0239) (Table 11 and Figure 6).

#### 3.4.3. Correlation Ultrasound Thicknesses and Neuropathy Disability Score (NDS)

**Figure 7:**
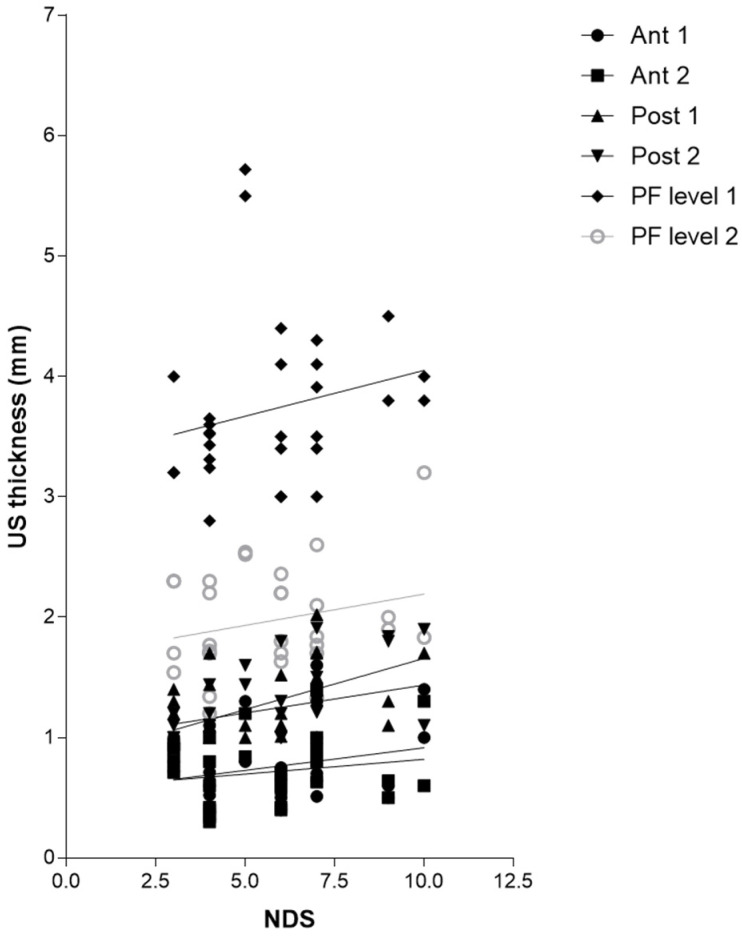
Correlation (Pearson R coefficient test) between fascial Ultrasound thickness and NDS. Crural fascia: Ant1, Ant 2, Post 1, and Post 2; Plantar fascia: PF level 1 and PF level 2.

Regarding Table 12, there was no detected statistically significant correlation between the NDS and US thicknesses of Ant 1, Ant 2, Post1, PL level 1, and level 2, whereas Post 2 showed a statistically significant correlation with the NDS (r = 0.5779, *p* = 0.0008) (Table 12 and Figure 7).

### 3.5. Intra-Rater Reliability

In addition, the intra-reliability was reported as good and excellent. The results for the CF and PF were as follows: Ant 1 (group 1: ICC _3,k_: 0.92; 0.88–0.96: group 2: ICC _3,k_: 0.92; 0.88–0.96); Ant 2 (group 1: ICC _3,k_: 0.92; 0.88–0.96: group 2: ICC _3,k_: 0.92; 0.88–0.96); Post 1 (group 1: ICC _3,k_: 0.94; 0.90–0.98: group 2: ICC _3,k_: 0.92; 0.88–0.96); Post 2 (group 1: ICC _3,k_: 0.92; 0.88–0.96: group 2: ICC _3,k_: 0.92; 0.88–0.96); PF level 1 (group 1: ICC _3,k_: 0.92; 0.88–0.96: group 2: ICC _3,k_: 0.92; 0.88–0.96); and PF level 2 (group 1: ICC _3,k_: 0.92; 0.88–0.96: group 2: ICC _3,k_: 0.88; 0.85–0.90) (Table 13).

## 4. Discussion

Based on our current knowledge, this study may be stated as the first study detailing the CF and PF thicknesses in chronic Charcot diabetic foot patients compared with healthy volunteers. As has been reported by other studies examining PF, the PF was easily visualized in the longitudinal axis at the calcaneal insertion, appearing as a multilayer, linear, hyperechogenic layers below the subcutaneous tissue [49], while no study studied it at the level of the middle third of the sole of the foot. Moreover, for the first time, this study evaluated the CF in chronic Charcot diabetic foot patients.

The study’s primary aim was to investigate the differences in CF and PF thicknesses at different regions/levels in chronic Charcot diabetic foot patients compared with healthy volunteers. An analysis of our results on the CF and PF thicknesses showed that in group 1, in the posterior region of the leg at Post 1 and Post 2 levels of the CF, the latter was thicker than in group 2, showing statistical differences (Post 1: group 1 vs. group 2: *p* = 0.03; Post 2: group 1 vs. group 2: *p* = 0.03) (Table 9, Figure 2 and Figure 3).

Moreover, an analysis of our results on the PF showed that in chronic Charcot diabetic foot patients (group 1), at two different levels, it was thicker than group 2 (PF level 1: Group 1 vs. Group 2: *p* < 0.0001; PF level 2: Group 1 vs. Group 2: *p* < 0.0001) (Table 9 and Figure 2 and Figure 4).

In light of these findings, the CF and PF tend to be thicker in chronic Charcot diabetic foot patients. They remodeled over time in response to repetitive stresses and diabetes pathology [25]. An increase in the CF thickness leads to a reduction in the ankle’s range of motion (ROM) [33,49], limiting its mobility and altering the gait [50] and the load distribution on the foot [51]. Furthermore, the involvement of CF and PF in transmitting forces within the lower limb is crucial [29,33]. It is worth noting that these structures can easily undergo significant alterations in terms of their thickness, stiffness, and impaired movement. They tend to remodel themselves in debilitated tissue that has become dense and fibrotic due to the effects of AGEs’ action [25]. These findings provided further confirmation, as supported by previous research [50,52], that changes in tissue, particularly in the fasciae [25], occur at an early stage in the progression of diabetes. Abate et al. [52] reported that in 51 patients with DM2, diagnosed less than a year prior, compared to 18 healthy volunteers, early fascial tissue changes with microvascular complications. Giacomazzi et al. [50] demonstrated, in a population similar to that of our study, how the PF thickness at calcaneal insertion increases concurrently with the degree of impairment of the nervous structures of the foot. In addition, all of the patients in group 1 showed values of NDS and NSS scores consistent with the diagnosis of neuropathy. A total of 30.4% of chronic Charcot diabetic foot patients showed vasculopathy. Only 13% of patients were affected by DM1, whereas 47% of all patients required insulin therapy. These data are in line with the published data about this type of diabetic foot [8,9,10,11]. Additionally, Fede et al. [26] demonstrated that in females, “the fascia becomes enriched in collagen-I, with low hormone levels, becoming more rigid during menopause”. According to this evidence, the greater number of women in menopause in the two groups studied could cause further fascial remodeling.

Furthermore, the correlation between the years of diabetes and CF US thickness of the poster region of the leg, respectively, for Post 1 (r = 0.3875, *p* = 0.0344), for Post 2 (r = 0.5089, *p* = 0.0041) (Table 10 and Figure 3), and between Post 2 and NDS (r = 0.5779, *p* = 0.0008), could be explained by the fact that the proximal progression of diabetes leads to involvement of CF and the latter becomes densified/fibrotic, consequently increasing its thickness [25] and altering their proprioception, with fascia richly innervated [53]. These observations could be confirmed surgically by the effectiveness of release intervention at the level of the myotendinous junction of the medial gastrocnemius [54], which could work on two fronts: (1) to reduce the tension on the Achilles tendon; (2) to hold CF, not foreseeing the surgical incision of the latter. The results have also confirmed, as has been demonstrated by other previous studies [22,23], that PF US thickness has increased in diabetic patients at calcaneal insertion; while no study studied it at the level of the middle third of the sole of the foot, this study for the first time demonstrated that also at this level there is an increase in the PF thickness, confirming that diabetes affects the whole plantar fascia and fasciae [25]. Moreover, the negative correlation between HbA1c and PF level 2 (middle third of the plantar surface) (r = −0.4115, *p* = 0.0239) could be explained by the fact that HbA1c is a punctual estimate of the glycaemic trend over a limited period of time, while, conversely, the PF thickness provides a more extensive representation of the progress of the disease, as the fascia presents a degenerative process lasting for years, resulting in more stable than glycaemic control. The collapse of the plantar arch typical of chronic Charcot diabetic foot could lead to a distribution of the load, such as compromise of the plantar fascia, leading to progressive thinning [25]. US examination could be revealed as a crucial tool to follow up with the patient and to intercept and prevent the progressive changes of diabetes, being portable and economical imaging. The outcomes have affirmed, mirroring previous investigations, that there exists a dependable and commendable level of intra-rater reliability in the US assessment when evaluating the deep fascia. This is particularly true for sonographers who possess optimal technical expertise in US assessment and a profound understanding of fascial anatomy [34].

This work represents the initial investigation that we are aware of, aiming to analyze and compare the thickness of the CF and PF in various regions/levels using US imaging in individuals with chronic Charcot diabetic foot conditions and compare them with those of healthy volunteers. In the future, extensive longitudinal studies involving a substantial number of patients will contribute significantly to our understanding of the underlying mechanisms behind diverse thickness patterns. Furthermore, US examination has the potential to reveal early changes in the fascia that cannot be detected during regular clinical examinations. Ultimately, defining CF and PF thickness in different regions/levels among these patients would enable a more precise and targeted approach to treatments and therapies. The reduction in tensions generated by proximal alterations to the foot could lead to indirect benefits also distally, with potential improvement in the biomechanics of gait and reduction in pressure in non-physiological load points. All that could reduce the risk of the most dramatic diabetic foot complication, ulceration [25].

### Limitation of Study

The limited power of the study makes it impossible to statistically analyze the prevalence of the US findings and explain their possible causes, prognostic significance, and therapeutic implications. Additionally, the US examination of CF and PF morphology heavily relies on the skill of the sonographer and the proper setting of the US device. Furthermore, the non-differentiation by sex and the non-blinding do not allow for generalizing the results; a large blinded study would be necessary to better contribute to our knowledge of the pathophysiology of different thickness patterns.

## 5. Conclusions

The US permits an optimal visualization of the fascial layers in patients with chronic Charcot diabetic foot patients, opening the road for a more in-depth comprehension of fascial changes in chronic Charcot diabetic foot. In addition, it may reveal changes, not only in plantar fascia but also in crural fascia, not highlighted by normal clinical examination. Some of these changes still need to be investigated further as they have not been fully described yet. In summary, the findings of the study confirmed that in patients with chronic Charcot diabetic foot, the PF is thicker at both its insertion point in the calcaneus and its middle third. Additionally, the CF was found to be thicker in posterior regions/levels compared to healthy volunteers. The observed thickness patterns of CF and PF in these patients suggested abnormal remodeling of the fascia due to the presence of diabetes and biomechanical alterations.

## Figures and Tables

**Table 1:** Descriptive data of group 1 (Charcot diabetic foot patients) and group 2 (healthy volunteers).

Group 1 N.	Sex	Age (y.)	Weight (Kg)	Height (cm)	BMI (Kg/m^2^)	Group 2 N.	Sex	Age (y.)	Weight (Kg)	Height (cm)	BMI (Kg/m^2^)
1	M	70	77.5	165	28.47	1	M	67	90	175	29.39
2	F	75	66	155	27.47	2	F	61	70	156	28.76
3	F	60	75.5	162	28.77	3	F	50	76	168	26.92
4	F	72	90.5	170	31.32	4	F	69	54	158	21.63
5	M	57	99	182	29.89	5	F	61	55	154	23.19
6	M	68	108	179	33.71	6	M	58	80	172	27.04
7	M	55	77	170	26.64	7	F	61	48	152	20.78
8	F	74	76.5	157	31.04	8	F	52	60	163	22.58
9	F	56	70.3	170	24.33	9	F	50	60	160	23.44
10	M	67	139	183	41.51	10	F	64	74	162	28.2
11	M	57	88.4	175	28.87	11	M	62	100	180	30.86
12	M	66	64.5	144	31.11	12	F	57	53	153	22.64
13	F	55	85	173	28.4	13	M	64	75	168	26.57
14	M	56	90.5	170	31.32	14	F	60	51	157	20.69
15	M	74	78.5	180	24.22	15	F	57	62	171	21.2
	**Mean ± SD**	64.1 ± 7.8	85.8 ± 18.9	169.0 ± 10.9	29.80 ± 4.16	16	M	66	75	178	23.67
							**Mean ± SD**	59.9 ± 5.7	67.7 ± 14.8	164.2 ± 9.1	24.85 ± 3.36

**Table 2:** Descriptive data associated with diabetes of group 2 (healthy volunteers). HBP: hypertensive blood pressure. DR: diabetic retinopathy. DN: diabetic nephropathy. y: years.

N.	Type	Insulin-	Time (y.)	HbA1c	Charcot	HBP	DR	DN
		Therapy		(%)				
1	DM2	No	9	6.3	Bilateral	HBP	No	No
2	DM2	No	15	6.6	Bilateral	HBP	No	No
3	DM2	No	8	6.6	Bilateral	HBP	No	No
4	DM2	Si	9	7.6	Bilateral	HBP	Yes	Yes
5	DM2	No	7	6.3	Right	HBP	No	No
6	DM2	Yes	21	6.82	Left	HBP	Yes	Yes
7	DM1	Yes	47	7	Right	HBP	Yes	No
8	DM2	No	23	6.82	Bilateral	HBP	No	No
9	DM2	No	6	5.99	Right	Normal	Yes	No
10	DM2	Si	27	7.3	Left	HBP	Yes	Yes
11	DM2	No	11	6.4	Right	HBP	Yes	No
12	DM2	Yes	40	6.91	Left	HBP	No	No
13	DM1	Yes	20	7.7	Bilateral	HBP	No	No
14	DM2	Yes	20	10	Bilateral	HBP	Yes	No
15	DM2	No	12	8	Bilateral	Normal	No	No

**Table 3:** Descriptive data associated with diabetes of group 2 (healthy volunteers).

Parameter	Value
Type of Diabetes (%)	
DM1	2/15 (13%)
DM2	13/15 (87%)
Insulin Therapy (%)	
Yes	7/15 (47%)
No	8/15 (53%)
Duration of Diabetes (years)	18.33 ± 12.15
HbA1c %	7.09 ± 0.98
Chronic Charcot Diabetes Foot (%)	
Right	4/15 (27%)
Left	3/15 (20%)
Bilateral	8/15 (53%)
Diabetic Retinopathy (%)	7/15 (47%)
Diabetic Nephropathy (%)	3/15 (20%)

**Table 4:** Clinical features of Group 1 detected at clinical evaluation. NDS: neuropathy disability score. NSS: neuropathy symptoms score. SF-12: 12-item short-form survey. Smoke: number of cigarettes per day. Alcohol: number of glasses per day.

N.	ABI Dx	ABI Sx	NDS	NSS	SF-12	Smoke	Alcohol
1	0.85	0.92	6	4	111.77	0	2
2	0.93	1.09	6	7	66.42	0	0
3	0.97	1.03	3	7	94.58	0	0
4	0.96	0.96	7	8	70.55	0	0
5	1.07	1	7	8	37.27	10	2
6	0.9	0.83	4	5	93.24	0	0
7	0.85	0.93	9	6	75.67	0	0
8	1	1.08	4	8	83.11	0	0
9	0.97	1.4	5	7	91.17	0	0
10	0.79	0.67	10	0	87.17	0	0
11	1.06	1	4	6	87.13	0	0
12	0.87	0.84	7	7	101.51	0	0
13	0.92	0.92	3	6	66.94	0	1
14	0.97	0.85	4	5	83.1	0	0
15	1	0.86	6	3	108.79	0	0

**Table 5:** Clinical features of group 1 detected at clinical evaluation.

Parameter	Value
ABI	
Right	0.94 ± 0.08
Left	0.96 ± 0.16
**Vasculopathy (ABI < 0.90)**	
Right	4
Left	5
**Charcot Vasculopathy**	
Right	2
Left	5

**Table 6:** Clinical features of group 2 detected at clinical evaluation.

N	ABI Right	ABI LEFT	SF-12	Smoke	Alcohol
1	1	1.03	101.07	0	0
2	1	0.93	111.45	0	0
3	1.13	1.09	111.78	0	0
4	0.97	0.98	110.34	0	0
5	1	1	102.65	0	0
6	1	1	98.64	0	1
7	1	1	107.26	0	0
8	1.1	0.97	99.91	0	0
9	1	1	82.89	0	0
10	0.84	0.84	88.44	0	0
11	1.08	1.06	95.59	10	2
12	1	1	85.43	0	0
13	1.03	1	114.69	0	1
14	1.02	1	92.03	0	0
15	1	0.95	93.46	0	0
16	1	1	114.6	0	1

**Table 7:** Ultrasound thickness measurements of the crural and plantar fasciae in the chronic Charcot diabetic foot patients (group 1).

Group 1	Ant 1	Ant 2	Post 1	Post 2	FP (Level 1)	FP (Level 2)
Number of values	30	30	30	30	30	30
Mean	0.752	0.7127	1.235	1.289	3.72	1.966
Std. deviation	0.3361	0.2854	0.3117	0.3025	0.6737	0.4343
Std. error of Mean	0.0614	0.0521	0.0569	0.05523	0.123	0.0793
Lower 95% CI of mean	0.6265	0.6061	1.119	1.176	3.469	1.804
Upper 95% CI of mean	0.8775	0.8192	1.351	1.402	3.972	2.128
Coefficient of variation	44.70%	40.05%	25.24%	23.47%	18.11%	22.09%

**Table 8:** Ultrasound thickness measurements of the crural and plantar fasciae in the healthy volunteers (group 2).

Group 2	Ant 1	Ant 2	Post 1	Post 2	FP (Level 1)	FP (Level 2)
Number of values	32	32	32	32	32	32
Mean	0.7184	0.76	0.9681	1.023	1.78	1.028
Std. deviation	0.137	0.1428	0.1933	0.2664	0.5705	0.4194
Std. error of mean	0.0242	0.0252	0.0342	0.0471	0.1008	0.0741
Lower 95% CI of mean	0.669	0.7085	0.8984	0.9268	1.575	0.8769
Upper 95% CI of mean	0.7678	0.8115	1.038	1.119	1.986	1.179
Coefficient of variation	19.08%	18.79%	19.97%	26.05%	32.04%	40.79%

**Table 9:** Ultrasound thickness measurements comparison between groups 1 and 2. Bold: statistically significant differences.

Group 1 vs. Group 2	Mean Diff.	95.00% CI of Diff.	Significant?	*p*-Value
**Ant 1**	−0.03356	−0.2838 to 0.2166	No	0.9995
**Ant 2**	0.04733	−0.2029 to 0.2975	No	0.9968
**Post 1**	−0.2669	−0.5171 to −0.01667	Yes	**0.0299**
**Post 2**	−0.2659	−0.5161 to −0.01565	Yes	**0.0308**
**FP level 1**	−1.94	−2.190 to −1.690	Yes	**<0.0001**
**FP level 2**	−0.9382	−1.188 to −0.6880	Yes	**<0.0001**

**Table 10:** Correlation (Pearson R coefficient test) between ultrasound measurements of thicknesses and years of diabetes.

Pearson r	Years of Diabetes	Years of Diabetes
	vs.	vs.
	Post 1	Post 2
r	0.3875	0.5089
95% confidence interval	0.0316 to 0.6562	0.1820 to 0.7345
R squared	0.1501	0.259
*p*-value		
*p* (two-tailed)	0.0344	0.0041
*p*-value summary	*	**
Significant? (alpha = 0.05)	Yes	Yes
Number of XY pairs	30	30

*: *p* < 0.05; **: *p* < 0.01.

**Table 11:** Correlation (Pearson R coefficient test) between Ultrasound measurements of Thicknesses and HbA1c in the group 1.

Pearson r	HbA1c vs. PF Level 2
r	−0.4115
95% confidence interval	−0.6721 to −0.06010
R squared	0.1693
0	
*p*-value	
*p* (two-tailed)	0.0239
*p*-value summary	*
Significant? (alpha = 0.05)	Yes
0	
Number of XY Pairs	30

*: *p* < 0.05.

**Table 12:** Correlation (Pearson R coefficient test) between Ultrasound measurements of Thicknesses and NDS.

Pearson r	NDS vs. Post 2
r	
95% confidence interval	0.2749 to 0.7765
R squared	0.334
*p*-value	
*p* (two-tailed)	0.0008
*p*-value summary	***
Significant? (alpha = 0.05)	Yes
Number of XY Pairs	30

***: *p* < 0.001.

**Table 13:** Intra-rater reliability of the ultrasound fascial thicknesses measurements within different regions/levels of group 1 and group 2.

Region/Level	ICC
Group 1 Ant 1	0.92 (0.88–0.96)
Group 1 Ant 2	0.92 (0.88–0.96)
Group 1 Post 1	0.94 (0.90–0.98)
Group 1 Post 2	0.92 (0.88–0.96)
Group 1 PF level 1	0.92 (0.88–0.96)
Group 1 PF level 2	0.92 (0.88–0.96)
Group 2 Ant 1	0.92 (0.88–0.96)
Group 2 Ant 2	0.92 (0.88–0.96)
Group 2 Post 1	0.92 (0.88–0.96)
Group 2 Post 2	0.92 (0.88–0.96)
Group 2 PF level 1	0.92 (0.88–0.96)
Group 2 PF level 2	0.88 (0.85–0.90)

## Data Availability

The data presented in this study are available upon request from the corresponding author. The data are not publicly available due to privacy.
